# Midregional Proadrenomedullin Improves Risk Stratification beyond Surgical Risk Scores in Patients Undergoing Transcatheter Aortic Valve Replacement

**DOI:** 10.1371/journal.pone.0143761

**Published:** 2015-12-02

**Authors:** Adam Csordas, Fabian Nietlispach, Philipp Schuetz, Andreas Huber, Beat Müller, Francesco Maisano, Maurizio Taramasso, Igal Moarof, Slayman Obeid, Barbara E. Stähli, Martin Cahenzly, Ronald K. Binder, Christoph Liebetrau, Helge Möllmann, Won-Keun Kim, Christian Hamm, Thomas F. Lüscher

**Affiliations:** 1 Department of Cardiology, University Heart Center Zürich, Zürich, Switzerland; 2 Department of Internal Medicine and Cardiology, Kantonsspital Aarau, Aarau, Switzerland; 3 Department of Cardiovascular Surgery, University Heart Center Zürich, Zürich, Switzerland; 4 Department of Cardiology, Kerckhoff Heart Center, Bad Nauheim, Germany; University Hospital Medical Centre, GERMANY

## Abstract

**Background:**

Conventional surgical risk scores lack accuracy in risk stratification of patients undergoing transcatheter aortic valve replacement (TAVR).

Elevated levels of midregional proadrenomedullin (MR-proADM) levels are associated with adverse outcome not only in patients with manifest chronic disease states, but also in the general population.

**Objectives:**

We investigated the predictive value of MR-proADM for mortality in an unselected contemporary TAVR population.

**Methods:**

We prospectively included 153 patients suffering from severe aortic stenosis who underwent TAVR from September 2013 to August 2014. This population was compared to an external validation cohort of 205 patients with severe aortic stenosis undergoing TAVR. The primary endpoint was all cause mortality.

**Results:**

During a median follow-up of 258 days, 17 out of 153 patients who underwent TAVR died (11%). Patients with MR-proADM levels above the 75^th^ percentile (≥ 1.3 nmol/l) had higher mortality (31% vs. 4%, HR 8.9, 95% CI 3.0–26.0, P < 0.01), whereas patients with EuroSCORE II scores above the 75^th^ percentile (> 6.8) only showed a trend towards higher mortality (18% vs. 9%, HR 2.1, 95% CI 0.8–5.6, P = 0.13). The Harrell’s C-statistic was 0.58 (95% CI 0.45–0.82) for the EuroSCORE II, and consideration of baseline MR-proADM levels significantly improved discrimination (AUC = 0.84, 95% CI 0.71–0.92, P = 0.01). In bivariate analysis adjusted for EuroSCORE II, MR-proADM levels ≥1.3 nmol/l persisted as an independent predictor of mortality (HR 9.9, 95% CI (3.1–31.3), P <0.01) and improved the model’s net reclassification index (0.89, 95% CI (0.28–1.59). These results were confirmed in the independent validation cohort.

**Conclusions:**

Our study identified MR-proADM as a novel predictor of mortality in patients undergoing TAVR. In the future, MR-proADM should be added to the commonly used EuroSCORE II for better risk stratification of patients suffering from severe aortic stenosis.

## Introduction

Transcatheter aortic valve replacement (TAVR) has emerged as a treatment option for patients with aortic stenosis at prohibitive, high or intermediate surgical risk [1–3]. Although at present TAVR is performed with a low intra-procedural complication rate and very favorable 30-day outcome, TAVR-patients still face a high long-term mortality reaching up to 44% at 3 years. The majority of deaths appear to be related to co-morbidities, rather than procedural complications, highlighting the importance of optimal risk assessment of patients considered for TAVR [4–8]. While also lower risk patients are currently evaluated for TAVR, it remains difficult to identify those that will not benefit from TAVR. Today, the risk-benefit evaluation for TAVR is mainly done on the basis of clinical judgment and surgical scoring systems such as the EuroSCORE II [9]. However, these commonly used risk scores have failed to truly predict long-term outcome in patients undergoing TAVR [10, 11].

Novel biomarkers may be useful for the prognostication and initiation of tailored therapies in patients deemed for TAVR. In this regard, the utility of midregional pro-adrenomedullin (MR-proADM), the midregional precursor of adrenomedullin (ADM) is of interest as it is an indicator for disease severity in a wide spectrum of cardiovascular disease states as well as various non-cardiovascular diseases including sepsis, COPD, pneumonia and chronic renal insufficiency [12–16]. Physiologically, ADM exhibits natriuretic and vasodilatatory effects, and it is expressed in different tissues, where it acts both as an autocrine and paracrine mediator [17, 18]. MR-proADM is a prime marker for global hemodynamic stress and cardiovascular dysfunction, and is highly predictive for adverse outcomes in patients with heart failure [19]. Indeed, MR-proADM levels are associated with important cardiovascular risk factors in elderly patients [20], and predict mortality in the general population [21]. Moreover, in unselected patients presenting to an emergency department, the MR-proADM plasma levels are elevated in proportion to disease severity, and possess the unique ability to identify patients at risk for short-term mortality beyond clinical risk assessment [22].

Whether pre-procedural MR-proADM levels are predictive for outcome in an elderly patient population undergoing TAVR has not been explored to date. In the present study, we therefore investigated the utility of pre-procedural MR-proADM for risk stratification of patients undergoing TAVR beyond the EuroSCORE II.

## Materials and Methods

### Study population

Patients were eligible for the study if they were accepted by the Heart Team to undergo transfemoral TAVR at the University Heart Center Zurich. Patients were recruited between September 2013 and August 2014, after giving written informed consent. Exclusion criteria were age < 18 years and infections (CRP > 20 mg/L), such as urinary tract infections, endocarditis, pneumonia or wound infections.

A second cohort of 205 patients derived from the Kerckhoff Heart Center in Bad Nauheim, Germany served as an external validation sample.

Approval for the current study was obtained from the Ethics Committee of the University of Zurich, and by the local Ethics committee for the Kerckhoff Heart Center TAVR registry. An independent critical event committee adjudicated all events of the study cohort.

### Measurement of MR-proADM levels

EDTA plasma samples were collected, frozen at the study site at -80°C and shipped to the Department of Internal Medicine, Kantonsspital Aarau, Switzerland, where the specimens were subsequently stored at -80°C. Samples were also collected at postoperative days 1–3. Measurement of MR-proADM levels was carried out as described previously [23].

### Clinical endpoints

The primary clinical endpoint for this analysis was defined as all-cause mortality. Device success and early safety were calculated using the VARC-2 definitions (Valve Academic Research Consortium-2) [24]. Procedure-related complications including high-degree AV-block with the need for pacemaker implantation, and outcome as related to post-procedural changes in the level of MR-proADM and C-reactive protein (CRP) levels, were assessed as secondary endpoints.

### Statistical analyses

Baseline plasma MR-proADM levels were measured in all 153 patients undergoing TAVR at the University Heart Center Zuerich. In 121 patients, a complete set of samples at postoperative days 1–3 was available. In the validation cohort, biomarkers were measured at baseline in 205 patients.

Normal distribution was tested using the Kolmogorov-Smirnov test; for variables that significantly differed from normality, the Mann-Whitney U test was used. Discrete values are expressed as counts (percentages), continuous variables as means (SD) or medians (IQR), as appropriate. Patients were dichotomized at the upper (75^th^) quartile of baseline levels of MR-proADM. For modeling the shape function of hazard rates, a flexible Royston-Parmar (RP) regression model with 3 degress of freedom (dfs) was employed [25]. To account for time-dependent hazard rates, the grouping variable was included as a time-dependent covariate. To delineate the time course of biomarkers following TAVR, a linear mixed effects regression model with random intercepts was employed with determination of an interaction between group and time. External validation of an RP-derived prediction index (PI) was carried out by application of the PI to the validation cohort, and comparison of the predicted survival functions with observed mortality (Kaplan Meier estimates). The incremental prognostic utility of MR-proADM to the univariate EuroSCORE II-derived model was evaluated by calculating the integrated discrimination improvement (IDI) and the net reclassification improvement (NRI). The discriminative accuracy and model calibration were assessed by Harrell's C-statistic and the Hosmer-Lemeshow test, respectively. All tests were two tailed; p < .05 was considered significant. Stata (StataCorp. 2011. Stata Statistical Software: Release 13. College Station, TX: StataCorp LP) was used for the statistical analyses.

## Results

### Baseline characteristics and distribution of MR-proADM levels

Baseline characteristics of the two patient cohorts investigated are shown in Tables 1 and 2, respectively. In the derivation cohort, both balloon expandable (SAPIEN XT or Sapien 3, n = 94) or self-expandable prostheses (CoreValve, Medtronic, n = 46) were used; in 13 patients, an alternative type of valve was used. In the validation cohort, TAVR was performed using balloon expandable (Edwards SAPIEN, n = 53) or self-expandable prostheses (CoreValve, Medtronic, n = 119); in 33 patients, an alternative type of valve was used. TAVR was performed via the transfemoral route in both patient cohorts.

**Table 1:** Baseline characteristics of the derivation study population according to all-cause mortality and upper quartile of MR-proADM.

Variables	Total sample (n = 153)	Survived (n = 136)	Deceased (n = 17)	P Value	Baseline MR-proADM quartile 1–3 (< 1.3 nmol/L) (n = 114)	Baseline MR-proADM quartile 4 (≥ 1.3 nmol/L) (n = 39)	P Value
**Clinical characteristics**							
Age,yrs (median, IQR)	82 (78–86)	82 (77–75)	86 (82–88)	0.03	82 (77–85)	83 (78–88)	0.11
Male sex, n (%)	76 (50)	64 (47)	12 (70)	0.06	59 (52)	17 (44)	0.37
EuroSCORE II (median, IQR)	3.9 (2.2–6.8)	3.5 (2.0–6.48)	5.8 (3.3–9.0)	0.03	3.2 (1.9–4.7)	7.9(4.3–12.3)	< 0.01
Body mass index, kg/m^2^ (median, IQR)	27.2 (25–31)	27.2 (25–30)	25.1 (24–32)	0.59	27.2 (25–31)	27.3 (23–32)	0.72
Diabetes, n (%)	42 (28)	36 (27)	6 (35)	0.46	31 (28)	11 (28)	0.95
Hypertension	134 (88)	118 (87)	16 (94)	0.38	102 (90)	32 (82)	0.22
Atrial Fibrillation, n (%)	55 (36)	48 (36)	7 (41)	0.64	34 (30)	21 (54)	0.01
Previous SAVR, n (%)	7 (5)	6 (5)	1 (6)	0.79	4 (4)	3 (8)	0.28
Previous CABG, n (%)	18 (12)	15 (11)	3 (18)	0.43	9 (8)	9 (23)	0.01
Previous CAD, n (%)	81 (52)	68 (50)	13 (76)	0.04	58 (51)	23 (59)	0.40
Previous malignoma, n (%)	27 (18)	23 (17)	4 (23)	0.50	19 (17)	8 (21)	0.60
Previous MI, n (%)	20 (13)	16 (12)	4 (24)	0.17	13 (12)	7 (18)	0.30
Previous stroke, n (%)	18 (12)	15 (11)	3 (18)	0.43	14 (12)	4 (10)	0.72
Renal failure^1^, n (%)	90 (59)	76 (56)	14 (82)	0.03	57 (50)	33 (85)	<0.01
COPD^2^, n (%)	22 (15)	18 (13)	4 (25)	0.20	14 (12)	8 (21)	0.18
Peripheral arteriopathy, n (%)	40 (26)	31 (23)	9 (53)	0.01	28 (25)	12 (31)	0.44
Pacemaker, n (%)	7 (5)	7 (5)	0 (0)	0.33	4 (4)	3 (8)	0.28
NYHA functional class III/IV, n (%)	106 (70)	94 (70)	12 (71)	0.93	74 (65)	32 (82)	0.05
**Laboratory**							
Hemoglobin, g/dl (mean, SD)	121 (18.6)	122 (18.2)	112 (20.5)	0.04	124 (18)	110 (16.6)	<0.01
MR-proADM, nmol/L (median, IQR)	1.1 (0.9–1.4)	1.0 (0.9–1.3)	2.1 (1.2–2.5)	<0.01			
proBNP, ng/l (median, IQR)	2054 (983–4972)	1876 (959–4073)	4506 (1752–12802)	0.01	1501 (844–2895)	6251 (2274–11015)	<0.01
Creatinine,mol/L (median, IQR)	101 (79–122)	99 (78–116)	140 (104–165)	0.01	95 (76–109)	124 (105–162)	<0.01
**Echocardiography**							
LVEF < 50, n (%)	52 (34)	48 (35)	4 (24)	0.33	34 (30)	18 (46)	0.06
Mean gradient, mmHg (median, IQR)	42 (31–53)	41 (31–53)	45 (34–51)	0.77	43 (31–53)	37 (30–51)	0.28
Aortic valve area, cm^2^ (median, IQR)	0.7 (0.6–0.9)	0.7 (0.6–0.9)	0.7 (0.6–0.9)	0.86	0.7 (0.6–0.9)	0.7 (0.6–0.8)	0.36

^1^GFR<60ml/min.

^2^Long-term use of bronchodilatators or steroids.

Abbreviations: GFR = glomerular filtration rate, SAVR = surgical aortic valve replacement, CABG = coronary artery bypass grafting, MI = myocardial infarction, NYHA = New York Heart Association, COPD = chronic obstructive pulmonary disease, BMI = body mass index, CAD = coronary artery disease.

**Table 2:** Baseline characteristics of the external validation population and according to all-cause mortality and upper quartile of MR-proADM.

Variables	Total sample (n = 205)	Survived (n = 168)	Deceased (n = 37)	P Value	Baseline MR-proADM quartile 1–3 (< 1.1 nmol/L) (n = 152)	Baseline MR-proADM quartile 4 (≥ 1.1 nmol/L) (n = 53)	P Value
**Clinical characteristics**							
Age,yrs (median, IQR)	83 (80–86)	83 (80–86)	83 (80–87)	0.66	83 (80–86)	83 (80–87)	0.84
Male sex, n (%)	80 (39)	66 (39)	14 (38)	0.87	62 (41)	18 (34)	0.38
EuroSCORE II (median, IQR)	6.6 (3.4–10.6)	6 (3.3–10.3)	8.4 (4.2–14.4)	0.04	5.6 (2.9–9.8)	8.2 (5.7–12.6)	<0.01
Body mass index, kg/m^2^ (median, IQR)	27 (24–31)	27 (24–31)	26 (24–31)	0.72	26 (24–30)	30 (27–35)	<0.01
Diabetes, n (%)	65 (32)	49 (29)	16 (43)	0.09	39 (26)	26 (49)	<0.01
Hypertension	196 (96)	159 (95)	37 (100)	0.15	144 (95)	52 (98)	0.30
Atrial Fibrillation, n (%)	73 (36)	57 (34)	16 (43)	0.28	48 (31)	25 (47)	0.04
Previous SAVR, n (%)	4 (2)	4 (2)	0	0.34	4 (3)	0	0.23
Previous CABG, n (%)	27 (13)	21 (13)	6 (16)	0.54	19 (13)	8 (15)	0.63
Previous CAD, n (%)	124 (60)	102 (61)	22 (60)	0.88	88 (58)	36 (68)	0.19
Previous MI, n (%)	28 (14)	22 (13)	6 (16)	0.61	18 (12)	10 (19)	0.2
Previous stroke, n (%)	29 (14)	24 (14)	5 (14)	0.90	22 (14)	7 (13)	0.82
Renal failure^1^, n (%)	89 (43)	69 (41)	20 (54)	0.14	52 (34)	37 (70)	<0.01
COPD^2^, n (%)	43 (21)	32 (19)	11 (30)	0.14	36 (24)	7 (13)	0.10
Peripheral arteriopathy, n (%)	27 (13)	20 (12)	7 (19)	0.25	19 (13)	8 (15)	0.63
Pacemaker, n (%)	23 (11)	16 (10)	7 (19)	0.10	16 (11)	7 (13)	0.59
NYHA functional cIass III/IV, n (%)	190 (93)	155 (92)	35 (95)	0.62	137 (90)	53 (100)	0.01
**Laboratory**							
Hemoglobin, g/dl (mean, SD)	124 (14.5)	125.7 (14.2)	115.5 (13.6)	0.01	126.6 (13.2)	116.8 (16)	0.03
MR-proADM, nmol/L (median, IQR)	0.9 (0.7–1.1)	0.9 (0.7–1.1)	1.1 (0.8–1.9)	0.01			
Creatinine, mol/L (median, IQR)	88 (70.4–114.4)	88 (70.4–114.4)	83.6 (70.4–123.2)	0.90	79.2 (70–105.6)	110 (105.6–140.8)	0.01
**Echocardiography**							
LVEF < 50, n (%)	55 (27)	41 (24)	14 (38)	0.09	35 (23)	20 (37)	0.03
Mean gradient, mmHg (median, IQR)	43 (34–52)	43 (37–52)	42 (26–50)	0.36	44 (37–52)	41 (26–52.5)	0.16
Aortic valve area, cm^2^ (median, IQR)	0.6 (0.5–0.7)	0.6 (0.5–0.7)	0.7 (0.5–0.8)	0.12	0.6 (0.5–0.7)	0.7 (0.5–0.8)	0.10

^1^GFR<60ml/min.

^2^Long-term use of bronchodilatators or steroids.

Abbreviations: GFR = glomerular filtration rate, SAVR = surgical aortic valve replacement, CABG = coronary artery bypass grafting, MI = myocardial infarction, NYHA = New York Heart Association, COPD = chronic obstructive pulmonary disease, BMI = body mass index, CAD = coronary artery disease.

In the derivation cohort, patients had a median age of 82 years (78–86), 50% were male. Baseline levels of MR-proADM ranged from 0.3 nmol/L to 4.4 nmol/L, with a median of 1.1 nmol/L and 25^th^ and 75^th^ percentile values of 0.8 nmol/L and 1.3 nmol/L, respectively. Median baseline values of MR-proADM were significantly higher in patients who died during follow-up, and patients in the upper quartile of MR-proADM also experienced a higher rate of procedural complications in both cohorts of patients (Tables 1–4).

**Table 3:** Procedural complications of the derivation patient population according to all-cause mortality and upper quartile of MR-proADM.

Variables	All Patients (n = 153)	Survived (n = 136)	Deceased (n = 17)	P Value	Baseline MRproADM quartile 1–3 (< 1.3 nmol/L) (n = 114)	Baseline MR-proADM quartile 4 (≥ 1.3 nmol/L) (n = 39)	P Value
Device success, n (%)	146 (95)	135 (99)	11 (65)	< 0.01	112 (98)	34 (87)	< 0.01
Early safety, n (%)	143 (94)	133 (98)	10 (59)	< 0.01	110 (96)	33 (85)	0.01
Myocardial infarction, n (%)	1 (1)	1 (1)	0	0.72	1 (1)	0	0.55
Emergency sternotomy, n (%)	3 (2)	0	3 (18)	< 0.01	0	3 (8)	< 0.01
Major vascular complications, n (%)	3 (2)	0	3 (18)	< 0.01	0	3 (8)	< 0.01
Life-threatening bleeding, n (%)	3 (2)	0	3 (18)	< 0.01	0	3 (8)	< 0.01
All stroke (disabling and non-disabling), n (%)	2 (1)	1 (1)	1 (6)	0.07	0 (0)	2 (5)	0.01
Need for pacemaker, n (%)	31 (21)	30 (22)	1 (8)	0.22	20 (18)	11 (31)	0.07
Use of extracorporeal membrane oxygenation, n (%)	4 (3)	0	4 (24)	< 0.01	1 (1)	3 (8)	0.02

**Table 4:** Procedural complications of the external validation cohort according to all-cause mortality and upper quartile of MR-proADM.

Variables	Total sample (n = 205)	Survived (n = 168)	Deceased (n = 37)	p Value	Baseline MR-proADM quartile 1–3 (<1.1 nmol/L) (n = 152)	Baseline MR-proADM quartile 4 (≥ 1.1 nmol/L) (n = 53)	p Value
Device success, n (%)	184 (90)	152 (90)	32 (86)	0.46	138 (91)	46 (87)	0.40
Early Safety, n (%)	188 (92)	168 (100)	20 (54)	<0.01	144 (95)	44 (83)	<0.01
Myocardial infarction, n (%)	0	0	0		0	0	
Emergency sternotomy, n (%)	6 (3)	5 (3)	1 (3)	0.92	5 (3)	1 (2)	0.60
Major vascular complications, n (%)	13 (7)	12 (7)	1 (3)	0.31	9 (6)	4 (8)	0.67
Life-threatening bleeding, n (%)	15 (7)	13 (7)	2 (5)	0.62	13 (9)	2 (4)	0.25
All stroke (disabling and non-disabling), n (%)	6 (3)	4 (2)	2 (5)	0.32	4 (3)	2 (4)	0.67
Need for pacemaker, n (%)	40 (20)	36 (22)	4 (11)	0.12	26 (17)	14 (26)	0.16
Use of extracorporeal membrane oxygenation, n (%)	6 (3)	5 (3)	1 (3)	0.92	5 (3)	1 (2)	0.60

### Association between baseline MR-proADM levels and mortality

During a median follow-up of 258 days (IQR 169–443), 17 patients died (11%), whereof 7 patients died within the first 30 postinterventional days and 10 patients died beyond 30 days.

Patients belonging to the upper quartile of MR-proADM (≥ 1.3 nmol/L) experienced a significantly higher rate of death as compared to subjects in quartile 1–3 (31% vs. 4%, HR 8.9, CI 3.0–26.0, P < 0.01), and the association with risk of death was maintained after adjusting for baseline EuroSCORE II (HR 9.9, 95% CI 3.1–31.3, P < 0.01). If analyzed as a continuous variable and after adjustment for EuroSCORE II, progressively higher MR-proADM values were associated with increased risk of death (HR 3.3, 95% CI 2.1–5.0, P < 0.01).

Patients who died during follow up had higher EuroSCORE II compared with survivors (3.6 vs. 5.9, P = 0.03). However, EuroSCORE II did not predict death (HR 2.1, 95% CI (0.8–5.6), P = 0.13 for upper quartile) (Table 5).

**Table 5:** RP-proportional hazards model with 3 dfs. Discriminatory ability of biomarker-enhanced models compared to existing risk schemes (EuroSCORE II) and proBNP.

Variable	Hazard ratio (95% CI)	P-Value	C-index (95% CI)	C-index difference (95% CI, P value)
**Univariate**				
ESII (per 1 unit increase)	1.1 (0.9–1.1)	0.16	0.58 (0.45–0.82)	
ES II (uper quartile)	2.1 (0.8–5.6)	0.13		
MR-proADM, nmol/L (per 1 unit increase)	3.1 (2.2–4.6)	< 0.01	0.84 (0.71–0.92)	0.20 (0.13–0.32, P = 0.01)
MR-proADM (upper quartile)	8.9 (3.0–26.0)	< 0.01		
proBNP, ng/L (per 1 unit increase)	1.0 (1.0–1.1)	0.03	0.69 (0.60–0.83)	0.11 (-0.09–0.13, P = 0.74)
proBNP (upper quartile)	3.1 (1.2–8.4)	0.02		
**Bivariate (MR-proADM + ESII)**				
ESII (per 1 unit increase)	1.0 (0.9–1.1)	0.68		
ES II (uper quartile)	0.6 (0.2–2.4)	0.53		
MR-proADM, nmol/L (per 1 unit increase)	3.3 (2.1–5.0)	< 0.01		
MR-proADM (upper quartile)	9.9 (3.2–31.3)	< 0.01		
**Bivariate (MR-proADM + proBNP)**				
MR-proADM, nmol/L (per 1 unit increase)	6.6 (2.9–14.9)	< 0.01		
MR-proADM (upper quartile)	6.1 (1.8–19.7)	< 0.01		
proBNP, ng/L (per 1 unit increase)	0.9 (0.9–1.0)	0.05		
proBNP (upper quartile)	1.2 (0.3–4.0)	0.75		

**Fig 1:**
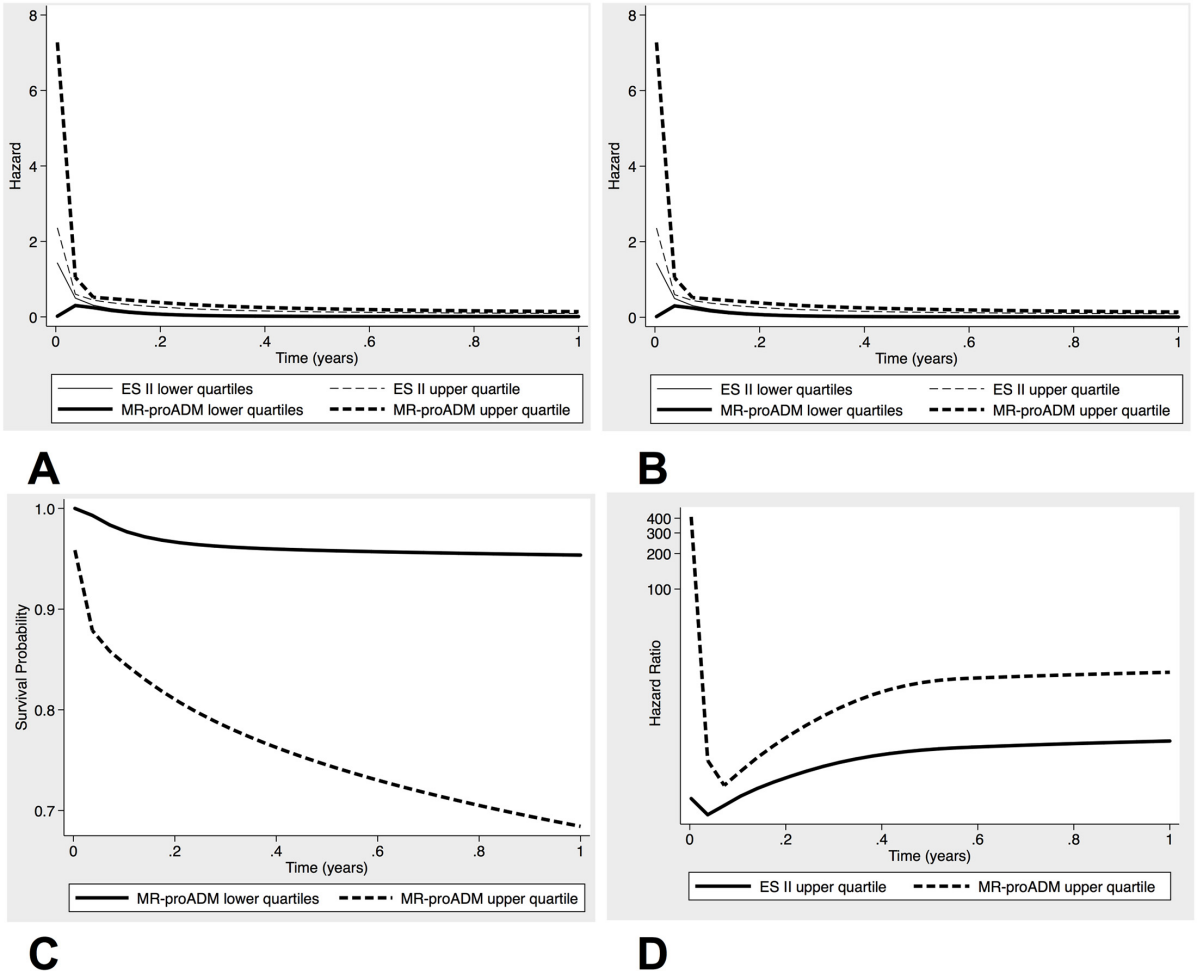
Mortality rates of patients receiving TAVR, grouped by upper quartile of MR-proADM and EuroSCORE II. (A) Hazard rates. (B) Cumulative hazard rates. (C) Cumulative survival probabilities. (D) Hazard ratio (ES II = EuroSCORE II).

As procedural mortality adds to the hazard rate during the early post-interventional phase, the baseline hazard function peaks early (Fig 1). For EuroSCORE II the AUC was 0.58 (95% CI, 0.45–0.82), and consideration of baseline MR-proADM levels significantly improved discrimination for mortality (AUC = 0.84 95% CI 0.71–0.92, P = 0.01). Baseline proBNP levels emerged as marginally significant predictors of mortality in the univariate analysis, but lost significance in conjunction with baseline MR-proADM values. Similarly, addition of proBNP to the EuroSCORE II did not significantly improve discrimination (Table 5).

### Relationship between time course of biomarkers and risk of death

**Fig 2:**
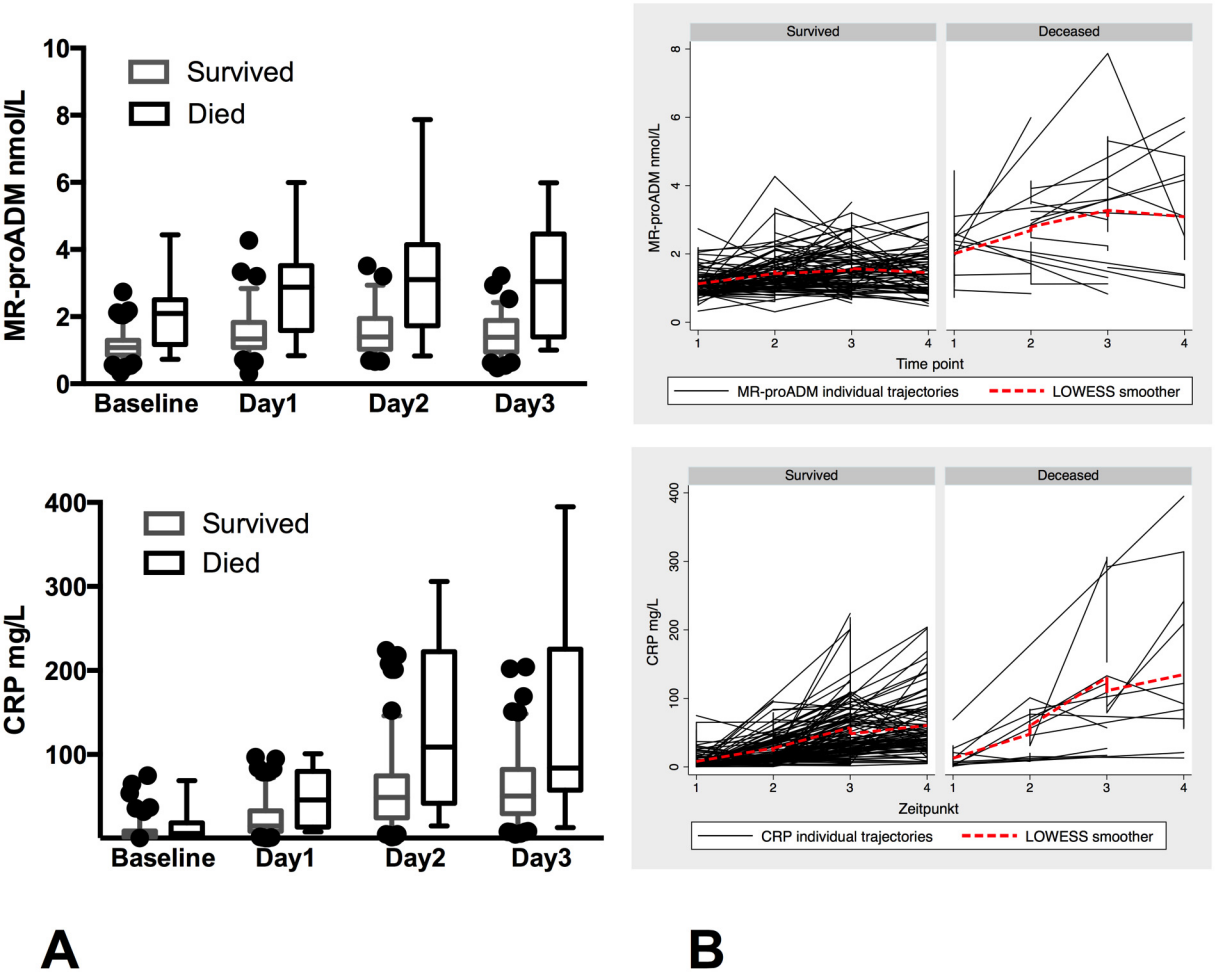
Longitudinal trajectories of MR-proADM and CRP at post-interventional day 1–3 following TAVR. (A) Median and interquartile range of biomarkers in patients after TAVR according to postoperative mortality. (B) Individual trajectories of MR-proADM and CRP with LOWESS smoothing following TAVR.

A significant increase of MR-proADM levels across all investigated time points was found (baseline/day1-3: P < 0.01; Fig 2). Similarly, CRP increased markedly during the early post-interventional period following TAVR (P < 0.01 for baseline/day1-3). MR-proADM showed a significant interaction between survivors and non-survivors at different time points (P_interaction_ < 0.01 for baseline/day 1–3). The same was true for CRP levels (P_interaction_ < 0.01 for baseline/day 1–3). No interaction was found with type of valve implanted and occurrence of post-interventional high-degree AV-block making pacemaker implantation necessary (P > 0.05 for all biomarkers investigated).

### External validation cohort

The baseline and procedural characteristics of the external validation cohort are shown in Tables 3 and 4. During a one-year follow-up, 37 patients died (18%). Baseline MR-proADM values ranged from 0.2 nmol/L to 4.3 nmol/L with a median of 0.9 nmol/L and 25th and 75th percentile values of 0.7 nmol/L and 1.1 nmol/L, respectively. Median MR-proADM levels were significantly higher in patients who died as compared to survivors (0.9 nmol/L vs. 1.1 nmol/L, P = 0.01). Belonging to the upper quartile of baseline MR-proADM was significantly associated with mortality in the univariate analysis (HR 3.9, 95% CI 1.6–5.6, P < 0.01), and after adjusting for EuroSCORE II (HR 2.7, 95% CI 1.4–5.1, P < 0.01).

### Discrimination, reclassification and validation of risk models

**Fig 3:**
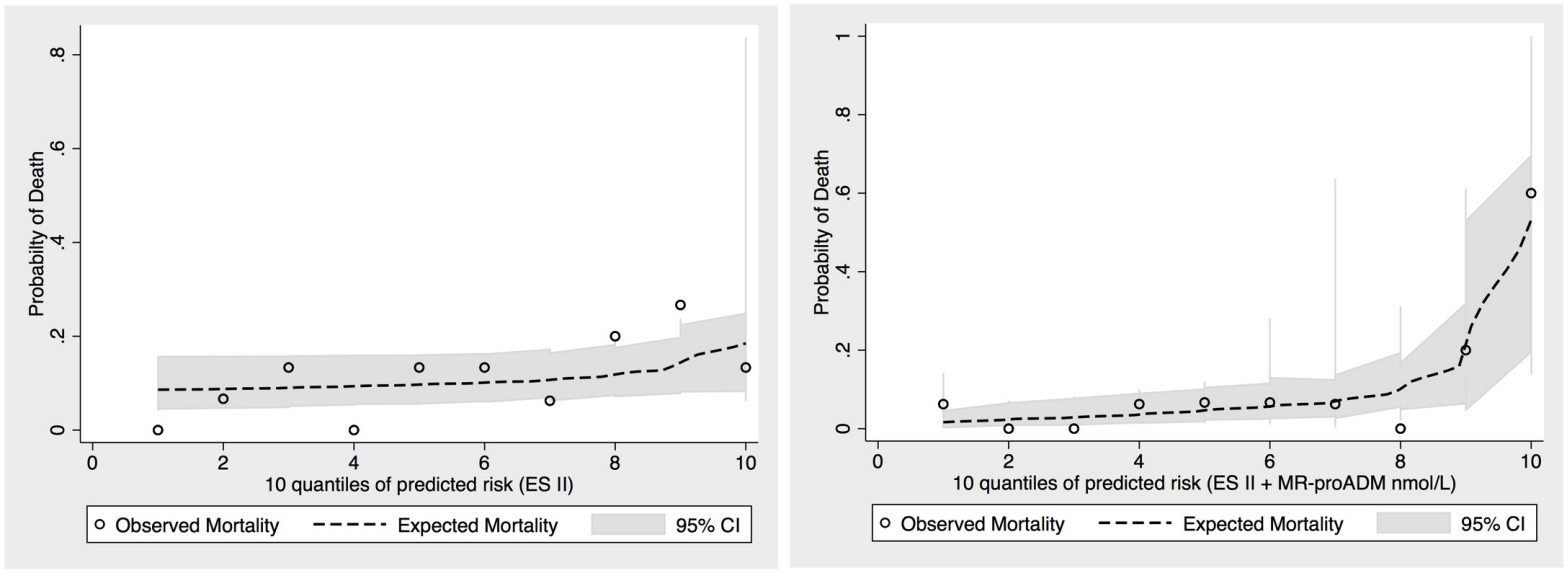
Predictiveness curves for the two risk models investigated (EuroSCORE II and EuroSCORE II+MR-proADM). Open circles display observed proportions of death within risk deciles. Left: model based on EuroSCORE II; right: model based on both EuroSCORE II and MR-proADM (both upper quartile) (ES II = EuroSCORE II).

**Fig 4:**
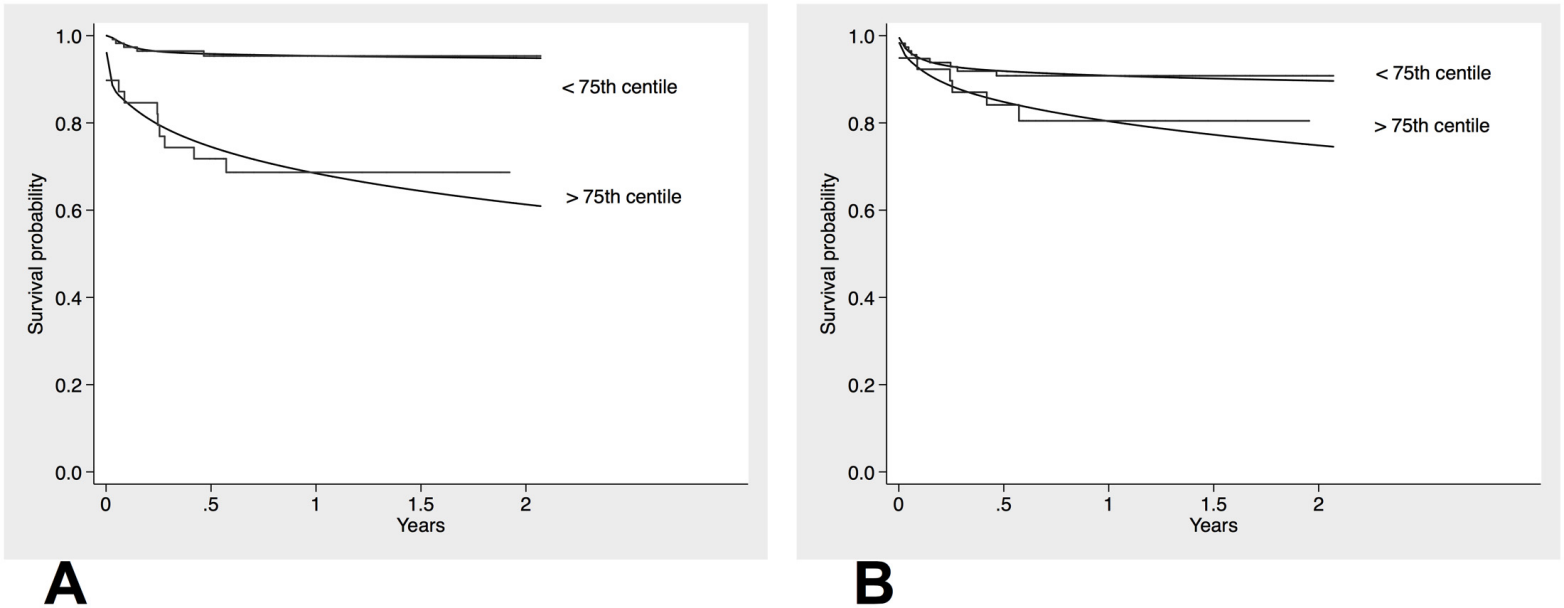
Overlay of Kaplan-Meier curves and RP mean survival curves in the two prognostic groups (upper and lower quartiles of RP-model predicted risk). (A) Model for MR-proADM + EuroSCORE II. (B) Model for EuroSCORE II.

**Fig 5:**
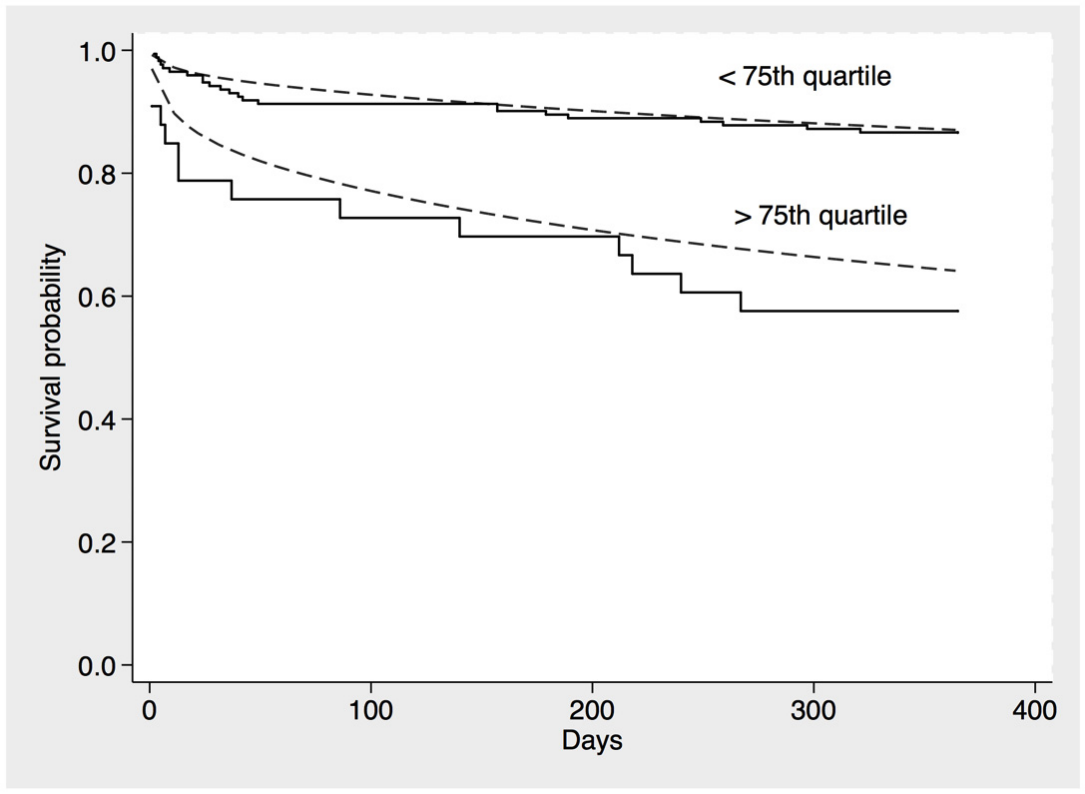
Validation of the RP-derived PI. **Solid lines: Kaplan-Meier survival curves in two prognostic groups (upper and lower quartiles of predicted risk) in the validation cohort.** Dashed lines: RP mean survival functions in the same prognostic groups of the validation cohort by the model fit to the derivation cohort.

**Fig 6:**
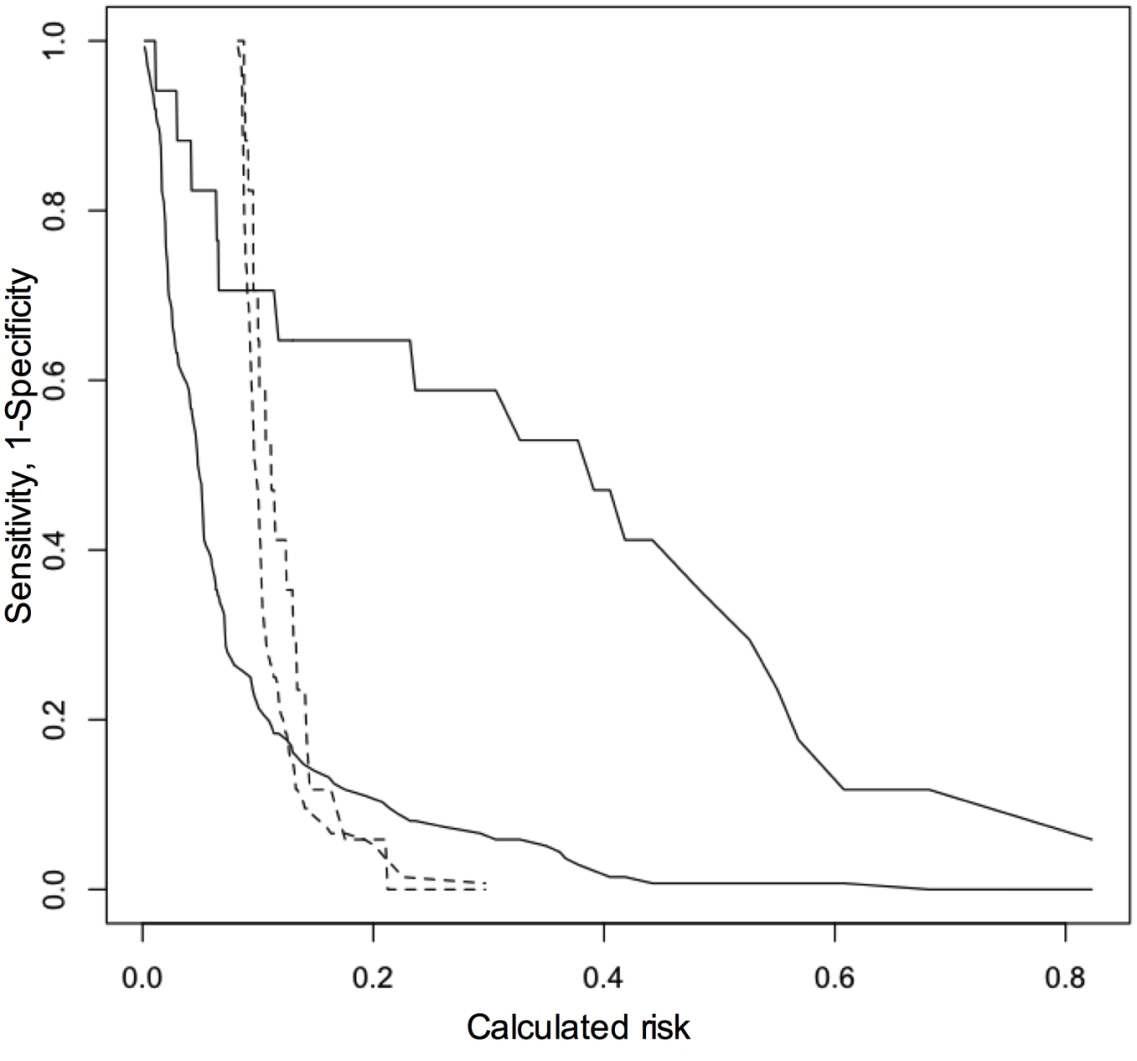
Clinical model enhancement by addition of MR-proADM. **Risk assessment plot for the reference (EuroSCORE II, dashed lines) and biomarker-enhanced (solid line) model for 1-year mortality;** 1-specificity versus calculated risk for those with the event and sensitivity versus calculated risk for those without events are shown.

To further establish the additive value MR-proADM levels in conjunction with the EuroSCORE II for predicting mortality, we established a univariate (EuroSCORE II) and bivariate (EuroSCORE II + MR-proADM) model for the binary outcome all-cause mortality, restricting analysis to patients who completed one-year follow-up. Adding MR-proADM values to the EuroSCORE II-based equation markedly improved reclassification for one-year mortality. Likelihood ratio testing confirmed a significantly enhanced model performance of an MR-proADM-based risk prediction algorithm (Table 6, Fig 3). We moved on to establish a prediction index and categorized the index into two prognostic groups (lower quartiles vs. upper quartile of predicted risk). While the biomarker-enhanced model enabled wide separation of prognoses among the subgroups, the EuroSCORE II-based model performed remarkably poor with regard to discrimination of patients according to outcome (Fig 4). Finally, we compared the predicted survival distributions form the derivation cohort with observed (Kaplan Meier) mortality in the validation cohort and observed reasonably well alignment between both curves (Fig 5). To further illustrate the diagnostic performance across risk thresholds of the univariate and bivariate model, we devised risk assessment plots for the reference and biomarker-enhanced model, and observed wide separation of both the event and nonevent curves between the models investigated (Fig 6).

**Table 6:** Reclassification and calibration indices of the MR-proADM-enhanced model as compared to commonly used risk schmes (EuroSCORE II).

Variable	Hosmer-Lemeshow goodness of fit (x^2^, P value)	LRT (x^2^, P value)	IDI events (95% CI)	IDI non-events (95% CI)	IDI (95% CI)	NRI events (95% CI)	NRI non-events (95% CI)	NRI (95% CI)	Risk category NRI events (95% CI)	Risk category NRI non-events (95% CI)	Risk category NRI (95% CI)
**MR-proADM nmol/L**	5.3, 0.7	29.8, <0.01	0.24 (0.07–0.45)	0.03 (0.01–0.97)	0.27 (0.07–0.51)	0.29 (-0.19 0.73)	0.60 (0.47–0.86)	0.89 (0.28–1.59)	-0.11 (-0.35–0.54)	0.53 (0.25–0.87)	0.41 (0.21–1.13)

Abbreviations: LRT = liklehood ratio test, IDI = integrated discrimination improvement, NRI = net reclassification index.

## Discussion

In the present study we evaluated the ability of MR-proADM to predict outcome in an unselected contemporary cohort of patients undergoing TAVR. We here for the first time report that higher baseline MR-proADM levels predict mortality following TAVR beyond of the commonly used EuroSCORE II, and this finding of the derivation cohort was confirmed in an independent validation cohort.

By adding MR-proADM into the equation, discrimination for mortality was markedly improved. Moreover, considering MR-proADM for risk assessement improved sensitivity and specificity for the clinical endpoint investigated across the calculated risk thresholds. Consequently, the predictiveness curve of the biomarker-enhanced model shows a much steeper slope at the high the risk spectrum than the univariate model, and its predictions align significantly better with the observed mortality rate as compared to the EuroSCORE II-based model. Although surgical risk scores consider several comorbidities, in the present study and in line with clinical experience, its discriminatory power for mortality proved rather modest with an AUC of 0.58.

Studies on natriuretic peptides were among the first to demonstrate the utility of biomarkers monitoring the hemodynamic state for risk stratification in patients considered for TAVR [26–28]. Of note, in the present study, MR-proADM was superior to proBNP in predicting mortality. In fact, the incremental predictive value of proBNP, when added to the EuroSCORE II, appeared rather modest, and proBNP lost its predictive value when modeled concomitantly with MR-proADM. The putative mechanism by which MR-proADM contributes to adverse outcomes might relate to its role as an unspecific marker of the general health status [21]. More specifically, its association with overall hemodynamic stress and endothelial dysfunction and its upregulation by hypoxia, inflammatory cytokines or shear stress may help to explain its role in the link between heart failure and poor outcomes. Mean serum MR-proADM values in healthy individuals measure around 0.33 nmol/L, thus the median level of 1.1 nmol/L observed in the present study is beyond normal values [23]. This finding may in part reflect hemodynamic stress among individuals with severe aortic stenosis. However, it was still possible to stratify patients according to quartiles of MR-proADM into subgroups with markedly varying outcomes. This finding aligns well with previous studies in which prototypical biomarkers of heart failure performed poorly as discriminators of risk in patients with aortic stenosis [29]. By its stress-responsive expression in many tissues and organ systems [30], MR-proADM appears to be poised well as a multidimensional indicator of poor health and reduced life expectancy in a heterogenous elderly patient population. In this regard, increased MR-proADM levels (≥ 1.3 nmol/L) appear to flag the patient as being at risk for a lack of durable benefit following TAVR, highlighting the value of biomarkers as a companion diagnostic in the decision making process of whether a patient will benefit from TAVR or not. Future studies are needed to define the optimal clinical decision criteria based on calculated risk in terms of classifying patients into clinically meaningful risk categories. In particular, adequately powered studies need to evaluate the sensitivity and specificity of risk-based thresholds in larger cohorts. The present study provides a useful starting point for further comparison of MR-proADM-enhanced models with commonly used risk scoring algorithms with regard to their ability to improve therapeutic triage of patients according to expected long term benefit. The parametric survival model employed in this study enabled generation of smooth estimates of the underlying hazard and survival function. Future studies should incorporate longer timer frames allowing for a better understanding of vulnerable periods following TAVR in an elderly patient population.

### Limitations

The moderate sample size of the study limited the possibility to control for multiple baseline characteristics as possible confounders of the distribution of MR-proADM. Consequently, adequately powered larger scale studies are needed to confirm our findings. However, the confirmation of our results in an independent external patient population adds importantly to the validity of the conclusions reached. Secondly, although MR-proADM was also analyzed as a continuous variable, the cut points applied in the study were not *a priori* specified but based on calculation of quartiles of MR-proADM upon entry into the study. Future studies are needed to define the clinical decision tree and optimal cut points that signify a high-risk phenotype in a heterogeneous elderly patients population awaiting TAVR.

### Conclusions

This is the first study on the role of MR-proADM as a companion diagnostic tool for risk stratification of patients undergoing TAVR. Our data support a biomarker-guided strategy for the evaluation of patients as to their suitability for TAVR with respect to expected long-term benefit.
